# Evaluating the Effect of Endoscopic Sinus Surgery on Laryngeal Mucosa Stroboscopic Features

**Published:** 2018-05

**Authors:** Ebrahim Karimi, Akbar Bayat, Mohammad Reza Ghahari, Sara Rahavi-Ezabadi, Mehrdad Jafari

**Affiliations:** 1 *Otorhinolaryngology Research Center, Tehran University of Medical Sciences, Tehran, Iran.*

**Keywords:** Endoscopy, Erythema, Edema, Laryngoscopy, Nasal polyps, Nasal obstruction, Stroboscopy

## Abstract

**Introduction:**

The major presenting symptom of nasal polyps is nasal obstruction. The role of nasal obstruction in the genesis of laryngeal disorders is still unknown.

**Materials and Methods:**

The aim of this study was to evaluate laryngeal videostroboscopic changes after functional endoscopic sinus surgery (FESS) in patients with nasal polyposis. A longitudinal study was carried out from March 2012 to June 2013. Thirty patients with bilateral nasal polyposis who did not respond to maximum medical treatment and were candidates for FESS were recruited. Laryngeal videostroboscopy was performed before and 3 months after FESS. Glottic gap, true vocal cord (TVC) borders and pliability, false vocal cord (FVC) movement, laryngeal erythema and mucosal edema were documented.

**Results:**

Laryngeal erythema and TVC edema were significantly decreased after FESS. Laryngeal erythema was documented in 18 patients after a 3-month follow-up. Four patients (13.3%) showed mild-to-moderate TVC edema and 26 patients (86.7%) had normal TVC mucosa.

**Conclusion:**

The results of this study show that FESS has a significant impact on laryngeal videostroboscopic features including laryngeal erythema and TVC edema.

## Introduction

Nasal polyps are common benign lesions affecting 4% of the general population. Some studies have reported a nasal polyp prevalence as high as 40% (1,2). Common clinical presentations of nasal polyps are nasal obstruction, congestion, facial pain or pressure, nasal discharge, post-nasal drip, hyposmia and headache (3). It has been shown that sinuses play an important role in the resonance of the voice. Although existing pathologies such as nasal polyposis, chronic rhinosinusitis, velopharyngeal insufficiency or adenoid hypertrophy cause nasal resonance, the role of nasal obstruction and nasality in the occurrence of laryngeal disorders is unknown. Cecil et al. recorded changes in the acoustic parameters of the voice after endoscopic sinus surgery in patients with nasal polyposis (4). Studies have also reported on the importance of nasal obstruction in children with dysphonia (5,6). In a study by De Labio et al., the videostroboscopic examination of children with nasal obstruction of varied causes revealed that 58% of children had a build-up of secretion on the vocal folds, hyperemia, congestion, mucosal thickening and vocal nodules (6).Studies that have evaluated the effect of nasal obstruction on laryngeal features in patients with nasal polyposis are so scarce that the results are inconclusive. The aim of this study was to assess the effect of nasal obstruction on videostroboscopic features in patients with nasal polyposis and to evaluate the effect of functional endoscopic sinus surgery (FESS) on laryngeal features.

## Materials and Methods

This longitudinal study was fully approved by the research ethics committee of Tehran University of Medical Science (protocol number 2013040612907NI). Written informed consent was obtained from all participants.

This study included 30 patients referred to Amiralam Hospital for FESS from March 2012 to June 2013. Patients older than 18 years with bilateral nasal polyps who did not respond to maximum medical treatment were eligible for this study. Nasal polyposis was diagnosed through clinical examination with nasal endoscopy and sinus computed tomography scan. Patients received at least 3 months intranasal corticosteroid nasal spray (fluticasone, two puffs twice a day) and nasal irrigation with 0.9% saline (three times a day). A careful history was taken, and a thorough physical examination was performed for each of the patients (none of our cases reported any history of pulmonary problems). Furthermore, all patients were assessed by an anesthesiologist as a pre-operative routine, and there were no reports of pulmonary problems in any of the patients. Patients with a history of previous nasal, laryngeal or pharyngeal surgery, gastroesophageal reflux, or a drug history of inhalational steroids, tracheal intubation, true vocal cord (TVC) paralysis, benign lesions of vocal cords or any systemic condition with possible laryngeal involvement such as amyloidosis, sarcoidosis or rheumatologic diseases were excluded.

All patients were examined by the first author and the speech pathologist to exclude primary functional or organic laryngeal diseases. All patients underwent gastroesophageal endoscopy, and patients with gastroesophageal reflux were excluded from the study. Patients underwent laryngeal videostroboscopic evaluation before and 3 months after endoscopic sinus surgery. During a 3-month follow-up, patients received intranasal corticosteroid spray (fluticasone, two puffs twice a day) and nasal irrigation with 0.9% saline (three times a day).

Laryngeal videostroboscopic recordings were made using a Karl Storz Pulsar II LED videostroboscope system. Video recordings were repeated with the same equipment used for the first recording and all were viewed on the same monitor.The operations were performed by our rhinology team, who had no role in the laryngoscopic assessments. The images were obtained by a speech pathologist. The stroboscopic recordings were evaluated by the authors (EK and AB) using the criteria set out by Hirano and Bless and also Colton et al. (7,8). The authors were blinded regarding the pre- or post-operative images. At least three complete cycles of TVC vibration were evaluated. The examiners were blinded regarding the assignment of each recording to pre- or post-operation. Statistical analysis was performed using PASW statistics 18.0.0. The McNemar test was used to compare the variables before and after surgery. A P<0.05 was considered statistically significant.

## Results

The mean (standard deviation) age of patients was 42.2 ± 12.4 years. Nineteen patients (63%) were male. Video laryngoscopy showed normal TVC movement in all patients on pre- and post-operative examination. In 29 patients (96.7%), phase symmetry was regular, while in one patient (3.3%) it was irregular on pre- and post-operative evaluation (Table.1).

**Table 1:** **Videostroboscopic characteristics on pre- and post-operation evaluation**

**Videostroboscopic characteristics**		**Pre-operation**	**Post-operation**
Phase symmetry	Regular	29	29
	Irregular	1	1
Glottis closure	Complete	23	23
	Mild spindle gap	5	5
	Mild posterior gap	2	2
TVC borders	Smooth	23	25
	Bowed	5	5
	Swollen	2	0
TVC pliability	Normal	29	29
	Mild stiffness	1	1
FVC compression	None	28	28
	Mild	1	1
	Moderate	1	1
	Severe	0	0
Laryngeal erythema	None	2	12
	Mild	15	15
	Moderate	13	3
	Severe	0	0
Mucosal edema	None	2	26
	Mild-to-moderate	28	4
	Moderate-to-severe	0	0

**Fig 1:**
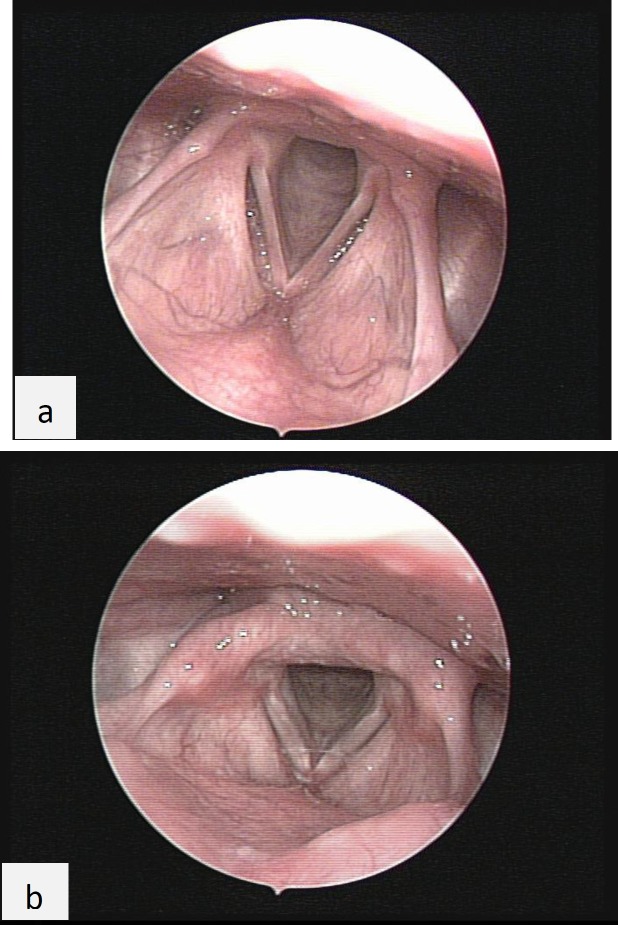
Laryngeal erythema before (a) and after (b) functional endoscopic sinus surgery

The results revealed that 23 patients (76.7%) had complete glottic closure during phonation and seven patients (23.3%) had mild glottic gap. Five patients had spindle gap and two patients had posterior gaps. TVC borders were smooth and straight in 23 patients (76.7%), bowed in five patients (16.7%) and swollen in two patients (6.7%) on videostroboscopic examination. TVC mucosal pliability was normal in 29 patients (96.7%) and mild stiffness was noticed in one patient (3.3%) on pre-operative evaluation. Twenty-eight patients had normal false vocal cord (FVC) movement, one patient (3.3%) showed mild FVC compression and one patient (3.3%) had moderate FVC compression on pre-operative examination. Right and left FVC compressions were the same in all patients. There were no statistically significant differences regarding glottis gap, TVC borders, TVC pliability or FVC movement between pre- and post-operation findings. There was no laryngeal erythema in two patients; mild erythema was seen in 15 and moderate erythema was seen in three patients. On post-operative evaluation, 12 patients had no laryngeal erythema, 15 patients had mild and three patients had moderate erythema. There was a statistically significant reduction in laryngeal erythema after FESS (Fig.1) (P=0.002).

**Fig 2:**
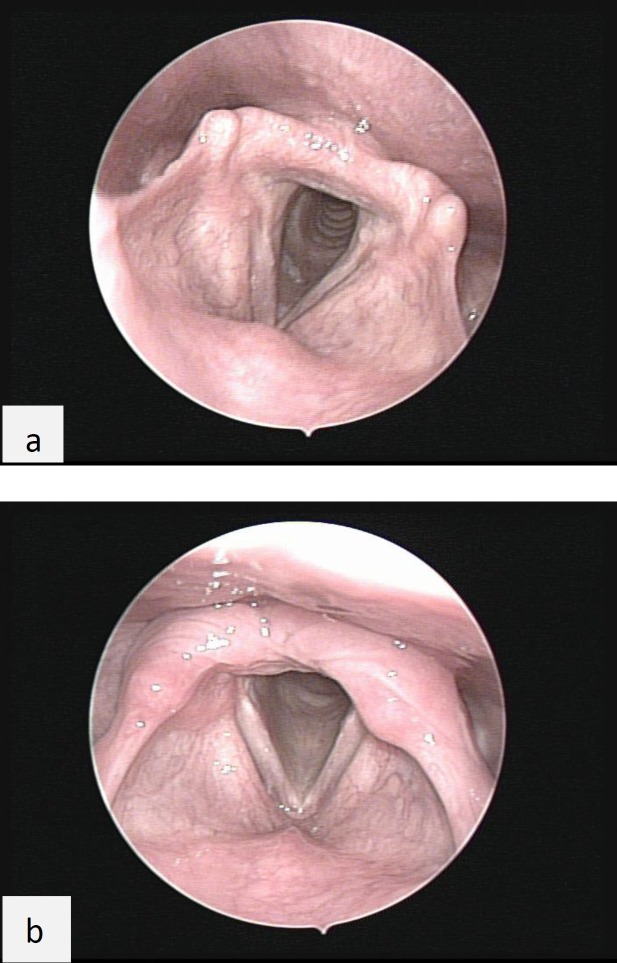
**Laryngeal edema before (a) and after (b) functional endoscopic sinus surgery**

We found that 28 patients (93.3%) had mild-to-moderate TVC mucosal edema and two patients (6.7%) had normal TVC mucosa. On post-operative evaluation, 26 patients (86.7%) had normal TVC mucosa and four patients (13.3%) showed mild-to-moderate TVC edema (Fig. 2). TVC edema was significantly improved after FESS (P<0.001).

## Discussion

Nasal polyp is considered a common benign disease, and most patients have long-term nasal congestion. Nasal obstruction may affect the laryngeal mucosa, but the true contribution of nasal obstruction in the pathogenesis of laryngeal disorders is still unknown. Some authors have reported the relationship between nasal obstruction and dysphonia. In a study conducted by De Labio et al., the effect of chronic nasal obstruction on laryngeal mucosa was investigated in pediatric patients (6). They found that 58% of patients had laryngeal changes, with mucosal thickening and laryngitis being the most common changes. Gomaa et al. evaluated the effect of adenoid hypertrophy on laryngeal changes, and reported laryngeal changes in 32.5% of patients (9). Meirelles examined 113 patients with vocal cord nodules, and reported nasal cavity changes including septal deviation, allergic rhinitis, nasal conchae bony changes, vasomotor rhinitis, tonsillar hypertrophy, nasal and antrochoanal polyposis (5). Mora et al. reported a marked improvement in acoustic vocal analysis and a reduction in dysphonic symptoms after tonsillectomy (10). Cecil et al. evaluated acoustic voice analysis in patients with nasal polyposis before and after FESS. They found that Jitter, Schimmer and noise-harmony ratio (NHR) values decreased and F_0_ value increased after FESS in patients with partial nasal obstruction (5).

The aim of this study was to evaluate the effect of nasal obstruction in patients with nasal polyposis on videostroboscopic laryngeal features by comparing laryngeal features before and after FESS. According to our review of literature, this is the first study that assesses the effect of nasal obstruction in nasal polyposis and compares the stroboscopic features before and after FESS. This study showed that laryngeal erythema and mucosal edema were the most common changes in patients with nasal polyposis, and proved FESS to have the potential for achieving a significant improvement in laryngeal erythema and mucosal edema.

Limitations of this study include the lack of a control group, and a relatively short follow-up period of only 3 months. To assess the long-term benefits, patients should be followed for a longer period of time, while applying outcomes such as voice quality and voice analysis help achieve a more comprehensive scope. Therefore, further studies including a control group are required to evaluate the consistency of our results.

## Conclusions

Although patients with nasal polyposis did not complain of laryngeal problems, some laryngeal mucosal abnormalities were evident in pre-operative laryngeal videostroboscopy. We found that laryngeal erythema and TVC edema were the most frequent videostroboscopic features of the larynx in patients suffering from nasal polyposis. Laryngeal edema and mucosal erythema could significantly improve after FESS in patients with nasal polyposis.
